# The complete mitochondrial genome of *Dunaliella salina* CS-265: insights into gene content and phylogenetic placement

**DOI:** 10.1080/23802359.2026.2635789

**Published:** 2026-03-02

**Authors:** A. K. Lisa, W. G. Reeve, D. W. Laird, A. Chopra, N. R. Moheimani

**Affiliations:** a School of Environmental and Conservation Sciences, Murdoch University, Murdoch, Australia; b Department of Biotechnology and Genetic Engineering, Gopalganj Science and Technology University, Gopalganj, Bangladesh; c School of Molecular and Life Sciences, Curtin University, Perth, Australia; d School of Mathematics, Statistics, Chemistry and Physics, Murdoch University, Murdoch, Australia; e IIID, Medical Genomics Core, Precision Medicine Centre, Murdoch University, Murdoch, Australia

**Keywords:** *Dunaliella salina*, mitochondrial genome, introns, gene loss, phylogeny, halotolerance

## Abstract

We report the complete mitochondrial genome of the halotolerant green alga *Dunaliella salina* CS-265, isolated from a hypersaline lake in central Australia. The genome is a circular DNA molecule of 30,073 bp, encoding seven protein-coding genes, nine rRNAs, and three tRNAs. Four core genes (*cox1*, *cob*, *nad1*, and *nad5*) are fragmented by multiple introns, whereas others remain intact. The absence of ATP synthase subunits and ribosomal protein genes reflects ongoing reductive evolution in *Dunaliella* mitochondria. This genome adds a new organellar resource from an Australian isolate, complementing previous studies and providing further insight into mitochondrial genome dynamics in halotolerant green algae.

## Introduction

*Dunaliella salina* Teodoresco 1905 is a halotolerant green alga widely recognized for its remarkable ability to survive in extreme saline conditions and for its capacity to produce high levels of β-carotene, particularly under environmental stress (Félix-Castro et al. 2023; Olmos 2024). These traits have positioned *D. salina* as a model organism for studying salt adaptation, oxidative stress response, and carotenoid biosynthesis in microalgae (Oren 2014).

While several mitochondrial genomes of *Dunaliella salina* have been published, including the Chilean strain CCM-UDEC 001 (KP691601, partial), the GN strain (KX641169), the Baja California strain SQ (KX641170), and the Western Australian Hutt Lagoon strain CCAP 19/18 (NC_012930), these sequences represent only a small fraction of the species’ global genetic diversity (Smith et al. 2010; Del Vasto et al. 2015; Magdaleno et al. 2017). Despite their environmental distinctness, their limited number, variable completeness, and inconsistent annotation quality highlight the need for more high-quality mitogenomes from well-characterized *D. salina* strains.

To address this, we present the complete mitochondrial genome of *D. salina* CS-265, isolated from a hypersaline lake in central Australia. This genome expands the available *Dunaliella* mitochondrial genome sequences, complementing previously published genomes and providing new context for comparative and evolutionary studies within the genus.

## Materials and methods

### Sample collection and culturing

*Dunaliella salina* CS-265 was obtained from the Australian National Algae Culture Collection (ANACC), CSIRO, Hobart, Australia (https://www.csiro.au/Research/Collections/ANACC). The strain was originally isolated by Murray Barton from Lake Suzie, Erldunda Station, Northern Territory, Australia (25.3385° S, 132.8270° E) on 1 January 1992 and is catalogued under voucher number CS-265 (contact: anacc@csiro.au).

Cultures were maintained in 3.5% F2 medium (Guillard 1975) at 25 °C (pH 7.5; 12:12 h light:dark) under 60 μmol photons m⁻^2^ s⁻^1^. For single-colony isolation, cells were plated on 1% agar F2 medium under 120 μmol photons m⁻^2^ s⁻^1^ and transferred to liquid culture for biomass propagation.

### DNA extraction and sequencing

Genomic DNA was extracted from stationary-phase cultures using the 2% CTAB method (Porebski et al. 1997). Whole-genome sequencing was carried out on a PromethION 2 platform (Oxford Nanopore Technologies, Oxford, UK) using the SQK-RBK114.24 kit and FLO-PRO114M flow cell (De Coster et al. 2018; Wang et al. 2021), with the assistance of the IIID, Medical Genomics Core Laboratory, Murdoch University, Murdoch, Australia. Basecalling was performed using the Super Accuracy mode in Dorado v0.7.4.14 (Oxford Nanopore Technologies 2023), the current standard ONT basecaller, to generate long-read data for mitochondrial genome assembly.

### Genome assembly and annotation

ONT long reads were mapped to three published *Dunaliella salina* mitochondrial genomes (KX641169, KX641170, NC_012930) using Minimap2 (Li 2018). Mapped reads were extracted, and duplicates were removed using Dedupe from the BBTools suite to eliminate redundancy (Bushnell 2014; Lantz et al. 2018). Error correction and normalization were performed using BBNorm (also from BBTools) to improve sequence quality and coverage uniformity (Bushnell 2014; Lantz et al. 2018). The curated dataset was assembled *de novo* using Flye v2.9.2 (Kolmogorov et al. 2019), resulting in a single circular contig of 30,073 bp with an average coverage of 197.07× and a GC content of 33.76% (Supplementary Figure S1). All genome mapping and assembly steps were conducted in Geneious Prime (v2025.1.2) (GraphPad Software LLC, La Jolla, CA). Assembly quality was assessed using QUAST v5.0.2 (Gurevich et al. 2013) via the Galaxy platform (The Galaxy Community 2022), using the *D. salina* GN mitochondrial genome (KX641169) as a reference. A detailed sequencing depth and coverage map is provided in Supplementary Figure S1.

Gene annotation was performed using GeSeq and annotation tools in Geneious Prime (GraphPad Software LLC 2025), guided by a reference *D. salina* mitochondrial genome (Tillich et al. 2017). Annotations were manually curated and validated with BLAST.

### Phylogenetic analysis

Phylogenetic relationships were inferred using a maximum-likelihood approach based on seven conserved mitochondrial protein-coding genes (PCGs) (*cob*, *cox1*, *nad1*, *nad2*, *nad4*, *nad5*, *nad6*) shared across 15 taxa, including a red algal outgroup. Genes were retrieved from GenBank, aligned with MAFFT (Katoh and Standley 2013), trimmed using trimAl (Capella-Gutiérrez et al. 2009), and concatenated. Maximum-likelihood analysis was performed in IQ-TREE v2.2.6 (Nguyen et al. 2015) under the GTR + F + G4 substitution model, which was automatically selected by the integrated ModelFinder algorithm (Kalyaanamoorthy et al. 2017).

### Synteny and genome rearrangement analysis

Comparative synteny was examined in progressiveMauve (Darling et al. 2004) by aligning the CS-265 mitochondrial genome with related *Dunaliella salina* genomes (KX641169, NC_012930).

## Results

**Figure 1.**
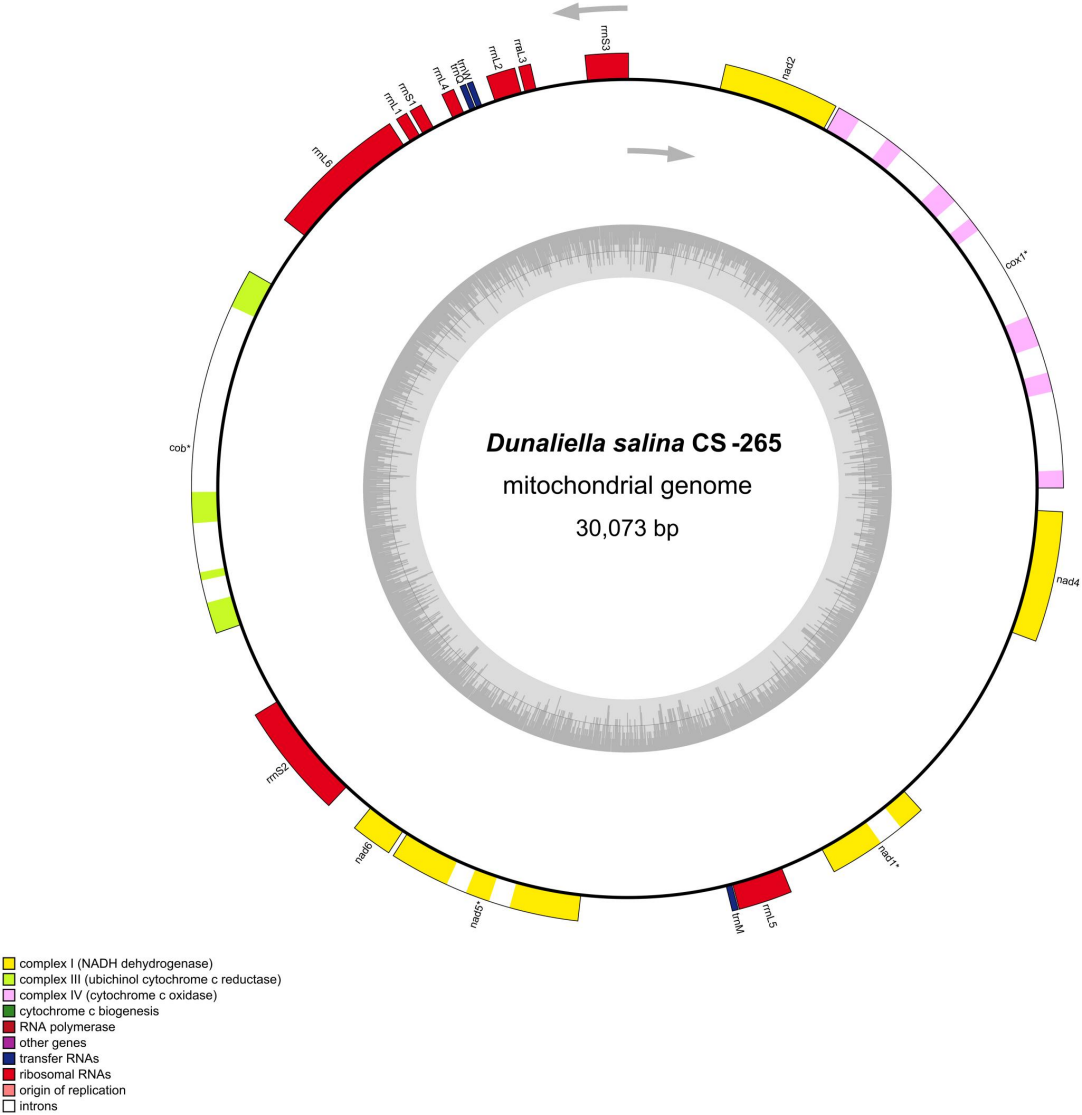
Mitochondrial genome map of *Dunaliella salina* strain CS-265. Circular representation of the complete mitochondrial genome of *D. salina* CS-265 generated using OGDRAW v1.3.1 (Greiner et al. 2019). Genes containing introns are indicated with an asterisk (*).

The complete mitochondrial genome of *Dunaliella salina* CS-265 is a circular DNA molecule of 30,073 bp (Figure 1) with an A + T bias of 66.24% (GC content 33.76%). Seven PCGs were identified, including five NADH dehydrogenase subunits (*nad1*, *nad2*, *nad4*, *nad5*, *nad6*) and two cytochrome genes (*cob* and *cox1*). Four genes (*cox1*, *cob*, *nad5*, *nad1*) contain introns and are divided across multiple exons: *cox1* (seven exons, six introns), *cob* (four exons, three introns), *nad5* (three exons, two introns), and *nad1* (two exons, one intron). The remaining genes are uninterrupted (*nad2*, *nad4*, *nad6*) (Supplementary Figure 2).

**Figure 2.**
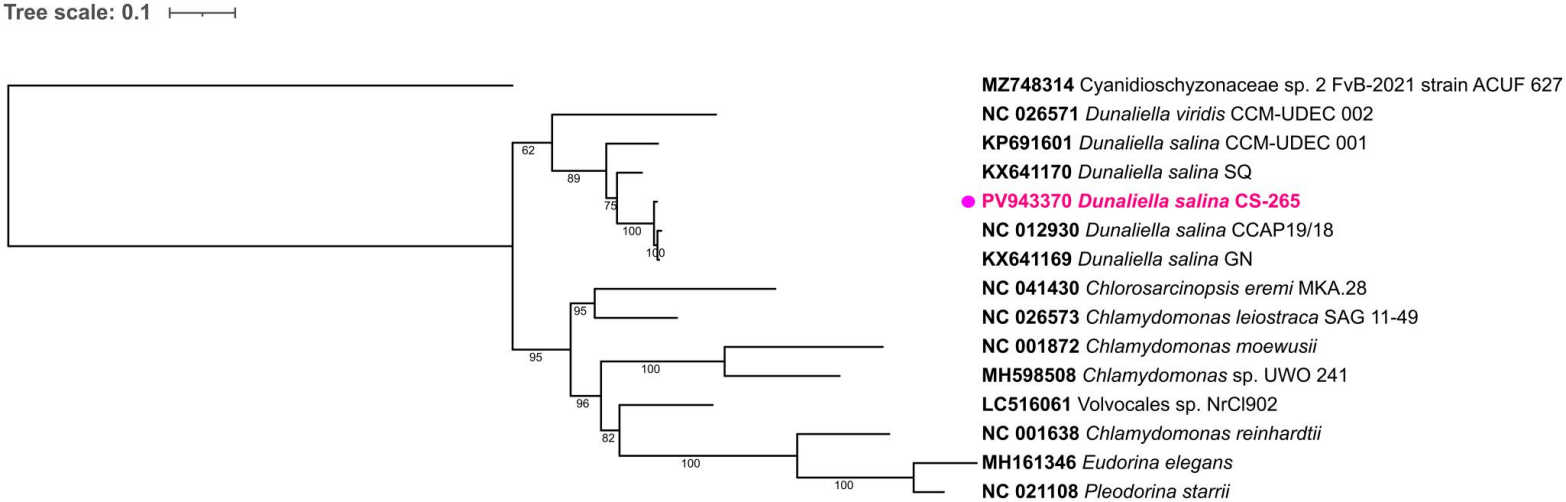
Phylogenetic tree of *Dunaliella salina* Teodoresco 1905 CS-265 and related taxa. The maximum-likelihood tree was constructed from concatenated mitochondrial protein-coding genes (*cob*, *cox1*, *nad1*, *nad2*, *nad4*, *nad5*, *nad6*). The newly assembled *D. salina CS-265* (highlighted in magenta) clusters within the *Dunaliella salina* clade. Bootstrap values (1000 replicates) are shown at the nodes. The following sequences were used: *Dunaliella salina* CCAP 19/18 (NC_012930; Smith et al. 2010), *D. salina* SQ (KX641170; Magdaleno et al. 2017), *D. viridis* CCM-UDEC 002 (NC_026571.1; Del Vasto et al. 2015), *D. salina* CCM-UDEC 001 (KP691601; Del Vasto et al. 2015), *D. salina GN* (KX641169; unpublished), *Chlamydomonas reinhardtii* (NC_001638.1; Vahrenholz et al. 1993), *Chlamydomonas moewusii* (NC_001872.1; Lee et al. 1991), *Chlamydomonas leiostraca* strain SAG 11-49 (NC_026573.1; Del Vasto et al. 2015), *Chlamydomonas* sp. UWO 241 (MH598508.1; unpublished), *Volvocales* sp. NrCl902 (LC516061.1; unpublished), *Pleodorina starrii* (NC_021108.1; Smith et al. 2013), *Eudorina elegans* (MH161346.1; unpublished), *Cyanidioschyzonaceae* sp. 2 FvB-2021 strain ACUF 627 (MZ748314.1; unpublished), and *Chlorosarcinopsis eremi* strain MKA.28 (NC_041430.1; unpublished).

The genome also encodes nine rRNA genes (including *rrnL* and *rrnS*, collectively >4000 bp) and three tRNAs – *trnM*, *trnQ*, and *trnW* (73–76 bp). The concatenated phylogenetic alignment was 7468 bp in length, including 3843 parsimony-informative sites. Phylogenetic analysis placed *Dunaliella salina* CS-265 within a well-supported *Dunaliella* clade, clustering with other *D. salina* mitogenomes and most closely with the SQ (KX641170) and CCAP 19/18 (NC_012930) strains (bootstrap ≥95%; Figure 2). *D. viridis* formed a separate but closely related lineage.

**Figure 3.**
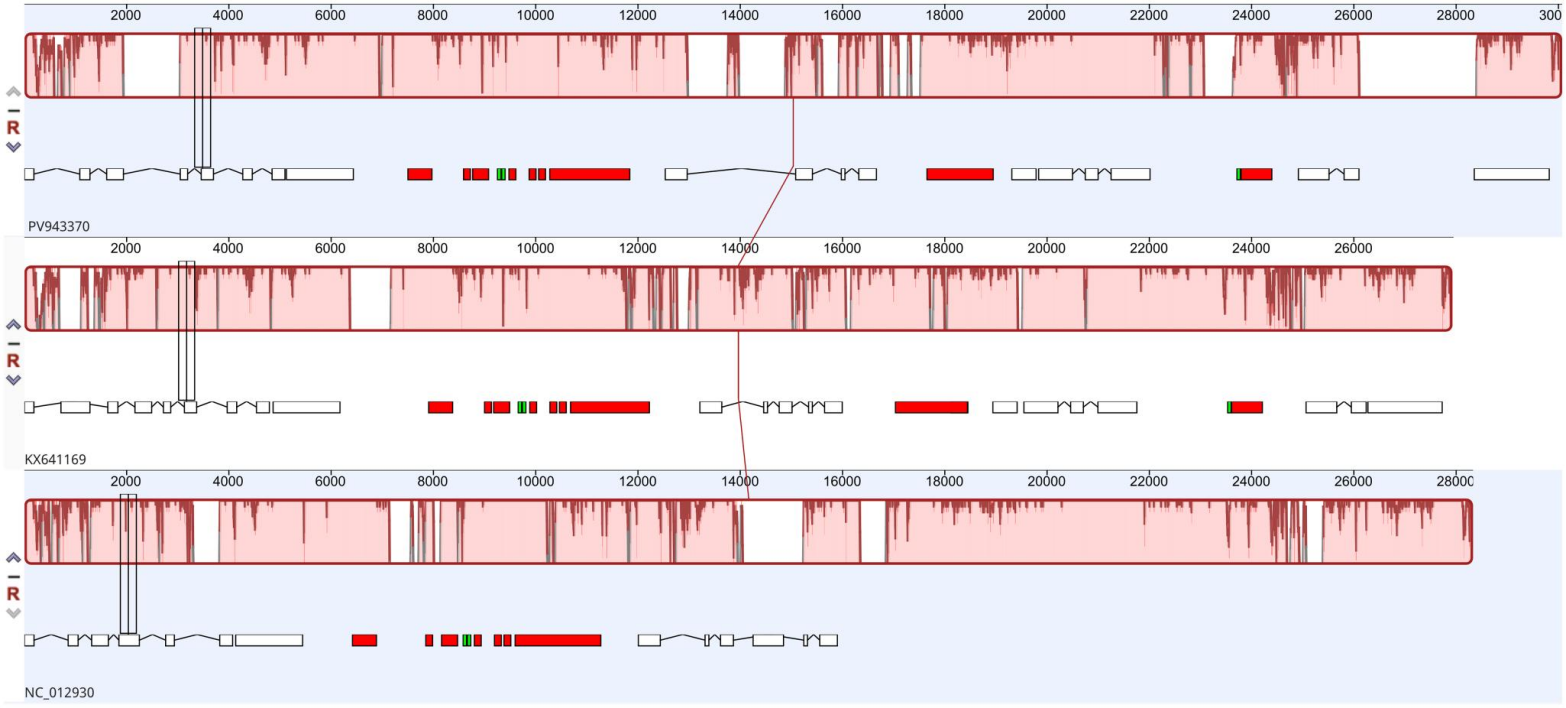
Mauve alignment of *Dunaliella salina* mitochondrial genomes. Whole-mitochondrial genome alignment of *D. salina* CS-265 (PV943370), *D. salina* GN (KX641169), and *D. salina* CCAP19/18 (NC_012930) showing conserved locally collinear blocks (LCBs) and structural rearrangements among strains.

Comparative synteny analysis showed that the CS-265 mitochondrial genome is largely collinear with the mitogenomes of closely related *D. salina* strains (CCAP19/18 and GN), exhibiting only small inversions and minor block shifts within conserved regions (Figure 3).

## Discussion

The mitochondrial genome of *Dunaliella salina* CS-265 is characterized by a compact architecture and a reduced gene set, consistent with mitochondrial streamlining observed across the Chlorophyta (Del Vasto et al. 2015; Smith and Keeling 2015). The marked A + T bias (66.24%) aligns with compositional trends reported in *Chlamydomonas reinhardtii* and other green algae, where organellar genomes are similarly dominated by A + T-rich regions (Smith et al. 2010; Massoz et al. 2014).

The presence of intron-rich genes – particularly *cox1*, *cob*, *nad1*, and *nad5* – reflects conserved patterns of gene fragmentation commonly mediated by group I and II introns in chlorophyte mitochondria (Smith et al. 2010; Fučíková et al. 2014; Del Vasto et al. 2015; Magdaleno et al. 2017). In contrast, intronless genes such as *nad2*, *nad4*, and *nad6* may indicate evolutionary intron loss or retention of structurally stable forms. This pattern is consistent across published *D. salina* genomes, except that the SQ strain retains a single intron in *nad4*. The absence of mitochondrial ATP synthase subunits and ribosomal proteins further supports gene reduction through endosymbiotic transfer or functional replacement by nuclear-encoded counterparts (Smith et al. 2010; Magdaleno et al. 2017).

The identification of only three tRNA genes suggests a reliance on cytosolic tRNA import or post-transcriptional editing, a strategy previously proposed for *Dunaliella* species (Smith et al. 2010; Smith and Keeling 2015; Magdaleno et al. 2017). Together, these features highlight a reductive and specialized mitochondrial genome, shaped by both evolutionary pressure and metabolic constraints in hypersaline environments.

## Conclusions

The mitochondrial genome of *Dunaliella salina* CS-265 adds new insight into the diversity and structural evolution of chlorophyte mitochondria. Its compact size, high A + T content, and intron-rich genes reflect key features of adaptation and genome reduction in halotolerant green algae.

## Supplementary Material

updated_Supplementary file.docx

Track changes.docx

## Data Availability

The complete mitochondrial genome of *Dunaliella salina* Teodoresco 1905 strain CS-265 has been deposited in the NCBI GenBank database under accession number PV943370, associated with BioSample SAMN49745146. The raw sequencing reads are available in the Sequence Read Archive (SRA) under accession number SRR35566088, linked to BioProject PRJNA1099393, and BioSample SAMN51783953.
