# Supplementary material for: The complete mitochondrial genome of Dunaliella salina CS-265: insights into gene content and phylogenetic placement
Source: Mitochondrial DNA B Resour. 2026 Mar 2;11(4):468–72. doi: 10.1080/23802359.2026.2635789 (PMC12954796; doi:10.1080/23802359.2026.2635789)
Supplement: updated_Supplementary file.docx [file TMDN_A_2635789_SM6127.docx]

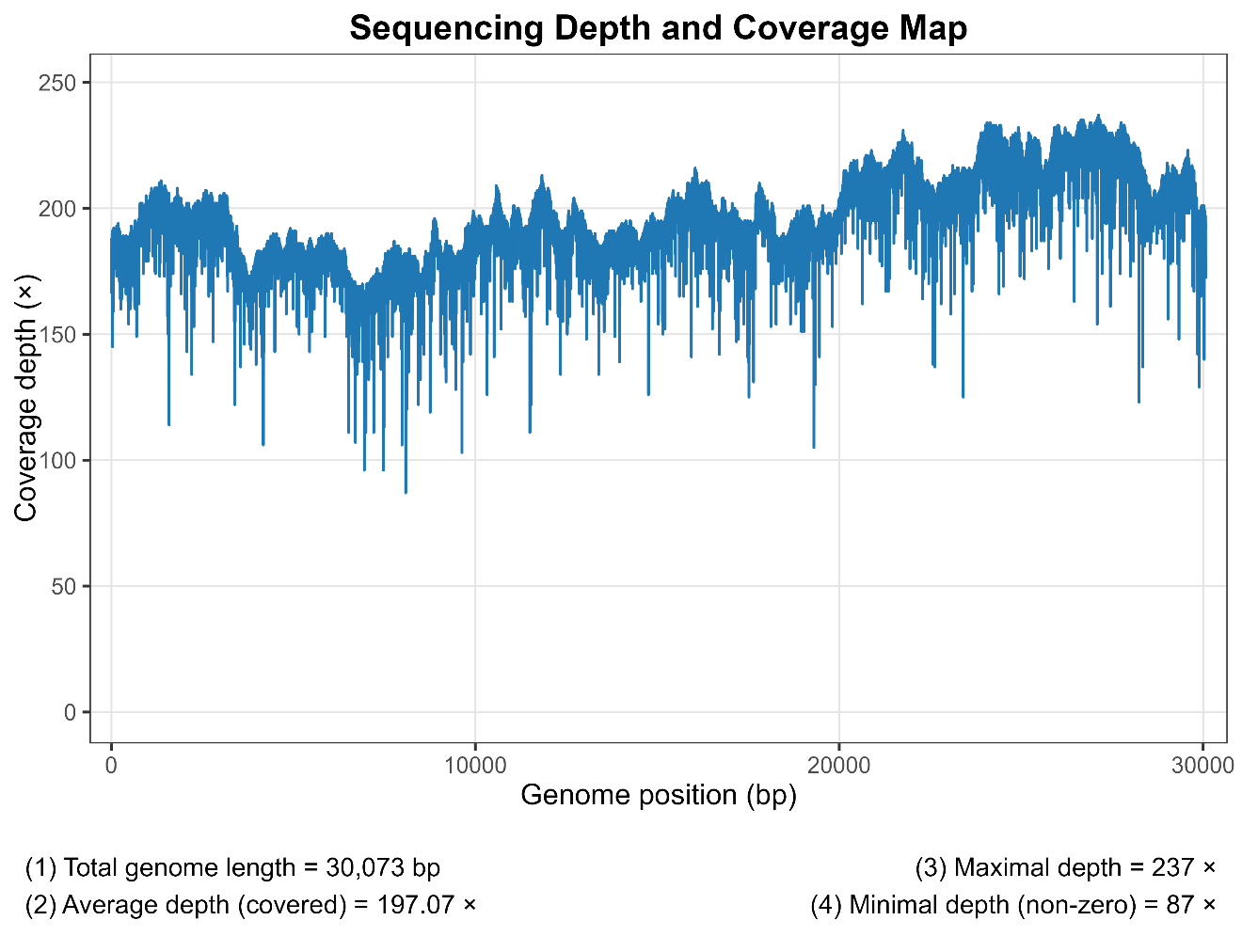


Figure S1: Sequencing depth and coverage map of the mitochondrial genome assembly of Dunaliella salina CS-265. The horizontal axis represents the base position of the mitogenome, and the vertical axis indicates the sequencing depth corresponding to each base.


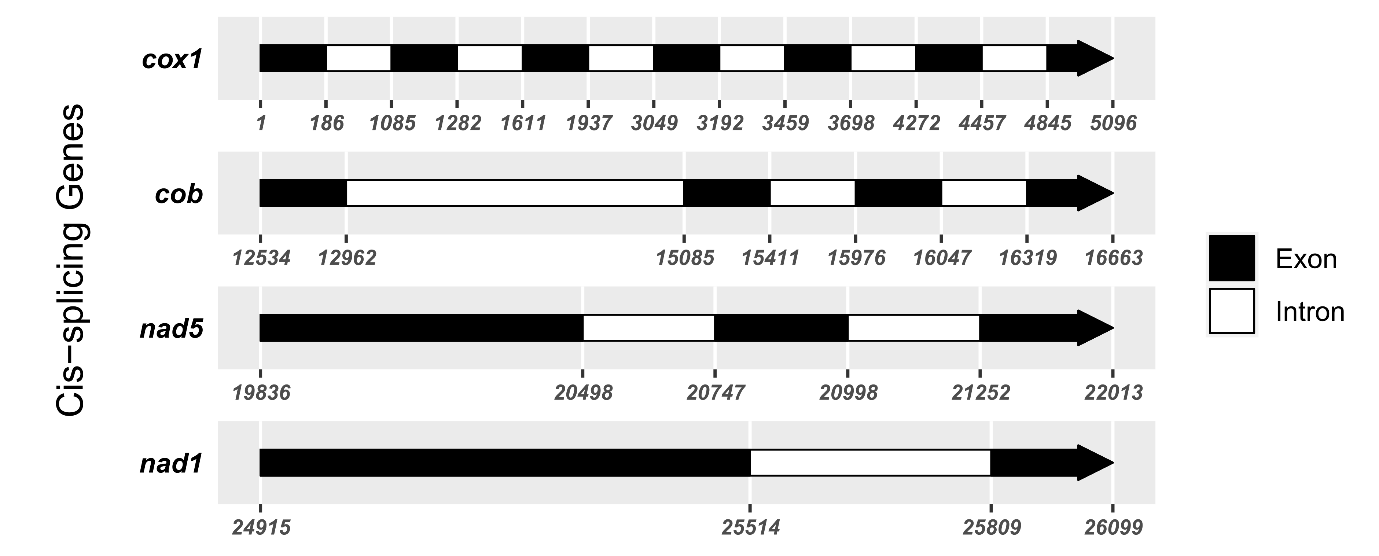


Figure S2: Schematic map of the cis-splicing genes in the mitochondrion genome of Dunaliella salina CS-265. Exons and introns are shown in black and white, respectively. The arrow indicates the sense direction of the gene.


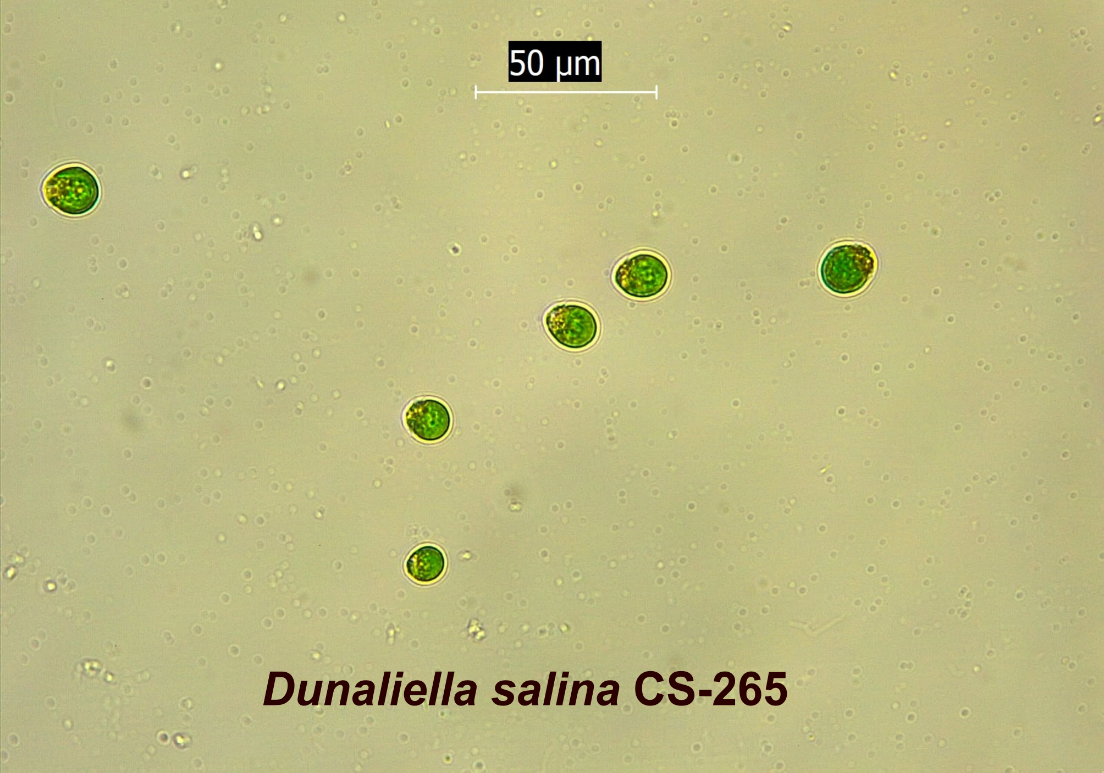


Figure S3: Light microscopy image of Dunaliella salina CS-265. Cells were imaged at 40× magnification. The image was obtained at the **Algae Innovation Hub, Murdoch University**. The cells are motile, green, and lack a rigid cell wall, traits typical of the genus. Scale bar = 50 µm.
