# Supplementary material for: The complete mitochondrial genome of Dunaliella salina CS-265: insights into gene content and phylogenetic placement
Source: Mitochondrial DNA B Resour. 2026 Mar 2;11(4):468–72. doi: 10.1080/23802359.2026.2635789 (PMC12954796; doi:10.1080/23802359.2026.2635789)
Supplement: Track changes.docx [file TMDN_A_2635789_SM6126.docx]

The Complete Mitochondrial Genome of *Dunaliella salina* CS-265: Insights into Gene Content and Phylogenetic Placement

Lisa AK^1,5^*, Reeve WG^2^, Laird DW^3^*, Chopra A^4^, Moheimani NR^1^

¹ School of Environmental and Conservation Sciences, Murdoch University, Western Australia

² School of Molecular and Life Sciences, Curtin University, Western Australia

³ School of Mathematics, Statistics, Chemistry and Physics, Murdoch University, Western Australia

⁴ IIID, Medical Genomics Core, Precision Medicine Centre, Murdoch University, Western Australia

⁵ Department of Biotechnology and Genetic Engineering, Gopalganj Science and Technology University, Gopalganj, Bangladesh

*Corresponding author: Lisa AK, School of Environmental and Conservation Sciences, Murdoch University, Perth, WA 6150, Australia.
Email: [33916617@student.murdoch.edu.au](mailto:33916617@student.murdoch.edu.au); personal: [asurabge@gmail.com](mailto:asurabge@gmail.com)

Laird DW, School of Mathematics, Statistics, Chemistry and Physics, Murdoch University, Western Australia
Email: [d.laird@murdoch.edu.au](mailto:d.laird@murdoch.edu.au)

# Abstract

# We report the complete mitochondrial genome of the halotolerant green alga *Dunaliella salina* CS-265, isolated from a hypersaline lake in central Australia. The genome is a circular DNA molecule of 30,073 bp, encoding seven protein-coding genes, nine rRNAs, and three tRNAs. Four core genes (*cox1*, *cob*, *nad1*, and *nad5*) are fragmented by multiple introns, whereas others remain intact. The absence of ATP synthase subunits and ribosomal protein genes reflects ongoing reductive evolution in *Dunaliella* mitochondria. This genome adds a new organellar resource from an Australian isolate, complementing previous studies and providing further insight into mitochondrial genome dynamics in halotolerant green algae.

# Keywords: *Dunaliella salina*, mitochondrial genome, introns, gene loss, phylogeny, halotolerance

# Introduction

*Dunaliella salina* Teodoresco 1905 is a halotolerant green alga widely recognized for its remarkable ability to survive in extreme saline conditions and for its capacity to produce high levels of β-carotene, particularly under environmental stress (Félix-Castro *et al*., 2023; Olmos, 2024). These traits have positioned *D. salina* as a model organism for studying salt adaptation, oxidative stress response, and carotenoid biosynthesis in microalgae (Oren, 2014).

While several mitochondrial genomes of *Dunaliella salina* have been published, including the Chilean strain CCM-UDEC 001 (KP691601, partial), the GN strain (KX641169), the Baja California strain SQ (KX641170), and the Western Australian Hutt Lagoon strain CCAP 19/18 (NC_012930), these sequences represent only a small fraction of the species' global genetic diversity (Del Vasto *et al.,* 2015; Magdaleno *et al.,* 2017; Smith *et al.,* 2010). Despite their environmental distinctness, their limited number, variable completeness, and inconsistent annotation quality highlight the need for more high-quality mitogenomes from well-characterised *D. salina* strains.

To address this, we present the complete mitochondrial genome of *D. salina* CS-265, isolated from a hypersaline lake in central Australia. This genome expands the available *Dunaliella* mitochondrial genome sequences, complementing previously published genomes and providing new context for comparative and evolutionary studies within the genus.

# Materials and Methods

## Sample Collection and Culturing

*Dunaliella salina* CS-265 was obtained from the Australian National Algae Culture Collection (ANACC), CSIRO, Australia (<https://www.csiro.au/Research/Collections/ANACC>). The strain was originally isolated by Murray Barton from Lake Suzie, Erlunda Station, Northern Territory, Australia (25.3385° S, 132.8270° E) on 1 January 1992 and is catalogued under voucher number CS-265 (contact: anacc@csiro.au).

Cultures were maintained in 3.5% F2 medium at 25 °C (pH 7.5; 12:12 h light:dark) under 60 μmol photons m⁻² s⁻¹. For single-colony isolation, cells were plated on 1% agar F2 medium under 120 μmol photons m⁻² s⁻¹ and transferred to liquid culture for biomass propagation.

## DNA Extraction and Sequencing

Genomic DNA was extracted from stationary-phase cultures using the 2% CTAB method (Porebski *et al.*, 1997). Whole-genome sequencing was carried out on a PromethION 2 platform (Oxford Nanopore Technologies) using the SQK-RBK114.24 kit and FLO-PRO114M flow cell (De Coster *et al.*, 2018; Wang *et al.*, 2021), with the assistance of the IIID, Medical Genomics Core Laboratory, Murdoch University. Basecalling was performed using the Super Accuracy mode in Dorado v0.7.4.14 (Oxford Nanopore Technologies, 2023), the current standard ONT basecaller, to generate long-read data for mitochondrial genome assembly.

## Genome Assembly and Annotation

ONT long reads were mapped to three published *Dunaliella salina* mitochondrial genomes (KX641169, KX641170, NC_012930) using Minimap2 (Li, 2018). Mapped reads were extracted, and duplicates were removed using Dedupe from the BBTools suite to eliminate redundancy (Bushnell, 2014; Lantz *et al.*, 2018). Error correction and normalization were performed using BBNorm (also from BBTools) to improve sequence quality and coverage uniformity (Bushnell, 2014; Lantz *et al.*, 2018). The curated dataset was assembled *de novo* using Flye v2.9.2 (Kolmogorov *et al.*, 2019), resulting in a single circular contig of 30,073 bp with an average coverage of 197.07× and a GC content of 33.76% (Supplementary Figure S1). All genome mapping and assembly steps were conducted in Geneious Prime (v2025.1.2) (GraphPad Software LLC). Assembly quality was assessed using QUAST v5.0.2 (Gurevich *et al.*, 2013) via the Galaxy platform (The Galaxy Community, 2022), using the *D. salina* GN mitochondrial genome (KX641169) as a reference. A detailed sequencing depth and coverage map is provided in Supplementary Figure S1.

Gene annotation was performed using GeSeq and annotation tools in Geneious Prime (GraphPad Software LLC, 2025), guided by a reference D. salina mitochondrial genome (Tillich et al., 2017). Annotations were manually curated and validated with BLAST.

## Phylogenetic Analysis

Phylogenetic relationships were inferred using a maximum-likelihood approach based on seven conserved mitochondrial protein-coding genes (*cob, cox1, nad1, nad2, nad4, nad5,* *nad6*) shared across 15 taxa, including a red algal outgroup. Genes were retrieved from GenBank, aligned with MAFFT (Katoh & Standley, 2013), trimmed using trimAl (Capella-Gutiérrez *et al.,* 2009), and concatenated. Maximum-likelihood analysis was performed in IQ-TREE v2.2.6 (Nguyen *et al.,* 2015) under the GTR+F+G4 substitution model, which was automatically selected by the integrated ModelFinder algorithm (Kalyaanamoorthy *et al.,* 2017).

## Synteny and Genome Rearrangement Analysis

Comparative synteny was examined in progressiveMauve by aligning the CS265 mitochondrial genome with related *Dunaliella salina* genomes (KX641169, NC_012930).

## Results

The complete mitochondrial genome of *Dunaliella salina* CS-265 is a circular DNA molecule of 30,073 bp (Figure 1) with an A+T bias of 66.24% (GC content 33.76%). Seven protein-coding genes (PCGs) were identified, including five NADH dehydrogenase subunits (*nad1*, *nad2*, *nad4*, *nad5*, *nad6*) and two cytochrome genes (*cob* and *cox1*). Four genes (*cox1*, *cob*, *nad5*, *nad1*) contain introns and are divided across multiple exons: *cox1* (7 exons, 6 introns), *cob* (4 exons, 3 introns), *nad5* (3 exons, 2 introns), and *nad1* (2 exons, 1 intron). The remaining genes are uninterrupted (*nad2*, *nad4*, *nad6*) (*Supplementary Figure 2*).

The genome also encodes nine rRNA genes (including *rrnL* and *rrnS*, collectively >4,000 bp) and three tRNAs—*trnM*, *trnQ*, and *trnW* (73–76 bp). The concatenated phylogenetic alignment was 7,468 bp in length, including 3,843 parsimony-informative sites. Phylogenetic analysis placed *Dunaliella salina* CS-265 within a well-supported *Dunaliella* clade, clustering with other *D. salina* mitogenomes and most closely with the SQ (KX641170) and CCAP 19/18 (NC_012930) strains (bootstrap ≥95%; Figure 2). *D. viridis* formed a separate but closely related lineage.

Comparative synteny analysis showed that the CS-265 mitochondrial genome is largely colinear with the mitogenomes of closely related *D. salina* strains (CCAP19/18 and GN), exhibiting only small inversions and minor block shifts within conserved regions (Figure 3).





Figure 1. Mitochondrial genome map of *Dunaliella salina* strain CS-265. Circular representation of the complete mitochondrial genome of *D. salina* CS-265 generated using OGDRAW v1.3.1 (Greiner *et al.*, 2019). Genes containing introns are indicated with an asterisk (*).


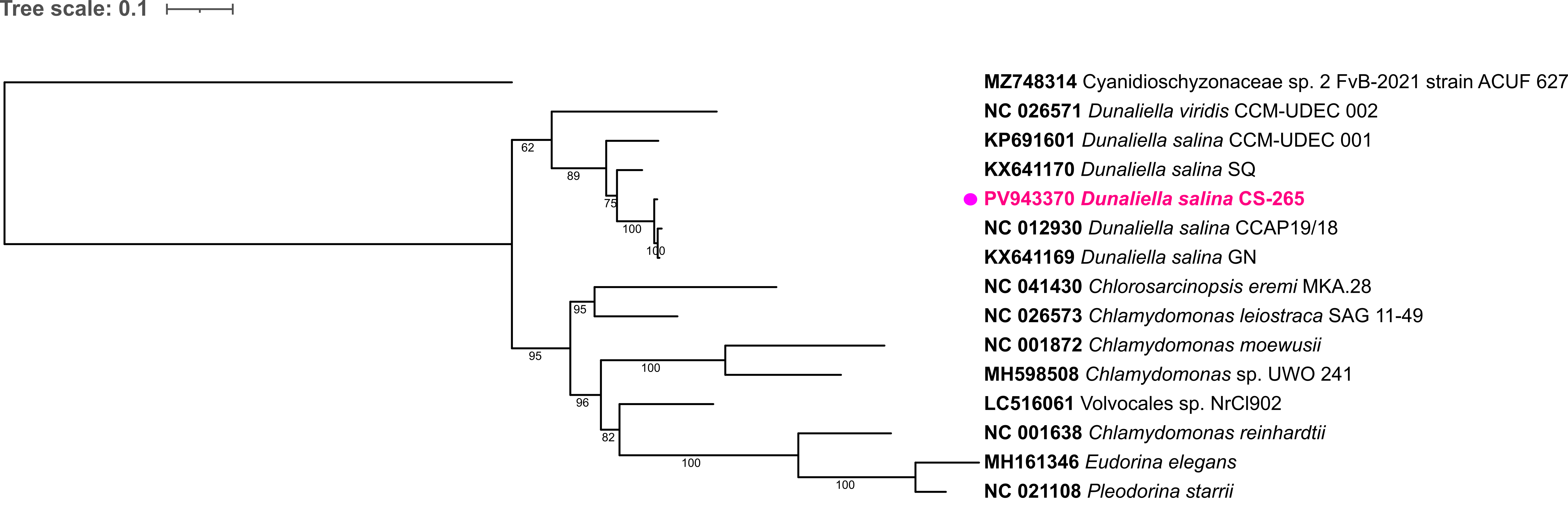


Figure 2. Phylogenetic tree of *Dunaliella salina* Teodoresco 1905 CS-265 and related taxa. The maximum likelihood tree was constructed from concatenated mitochondrial protein-coding genes (*cob, cox1, nad1, nad2, nad4, nad5, nad6*). The newly assembled *D. salina CS-265* (highlighted in magenta) clusters within the *Dunaliella salina* clade. Bootstrap values (1,000 replicates) are shown at the nodes. The following sequences were used: *Dunaliella salina* CCAP 19/18 (NC_012930; Smith *et al*., 2010), *D. salina* SQ (KX641170; Magdaleno *et al*., 2017), *D. viridis* CCM-UDEC 002 (NC_026571.1; Del Vasto *et al*., 2015), *D. salina* CCM-UDEC 001 (KP691601; Del Vasto et al., 2015), *D. salina GN* (KX641169; unpublished), *Chlamydomonas reinhardtii* (NC_001638.1; Vahrenholz *et al*., 1993), Chlamydomonas *moewusii* (NC_001872.1; Lee *et al*., 1998), *Chlamydomonas leiostraca* strain SAG 11-49 (NC_026573.1; Del Vasto *et al*., 2015), *Chlamydomonas* sp. UWO 241 (MH598508.1; unpublished), Volvocales sp. NrCl902 (LC516061.1; unpublished), *Pleodorina starrii* (NC_021108.1; Smith *et al*., 2013), *Eudorina elegans* (MH161346.1; unpublished), Cyanidioschyzonaceae sp. 2 FvB-2021 strain ACUF 627 (MZ748314.1; unpublished), and *Chlorosarcinopsis eremi* strain MKA.28 (NC_041430.1; unpublished).


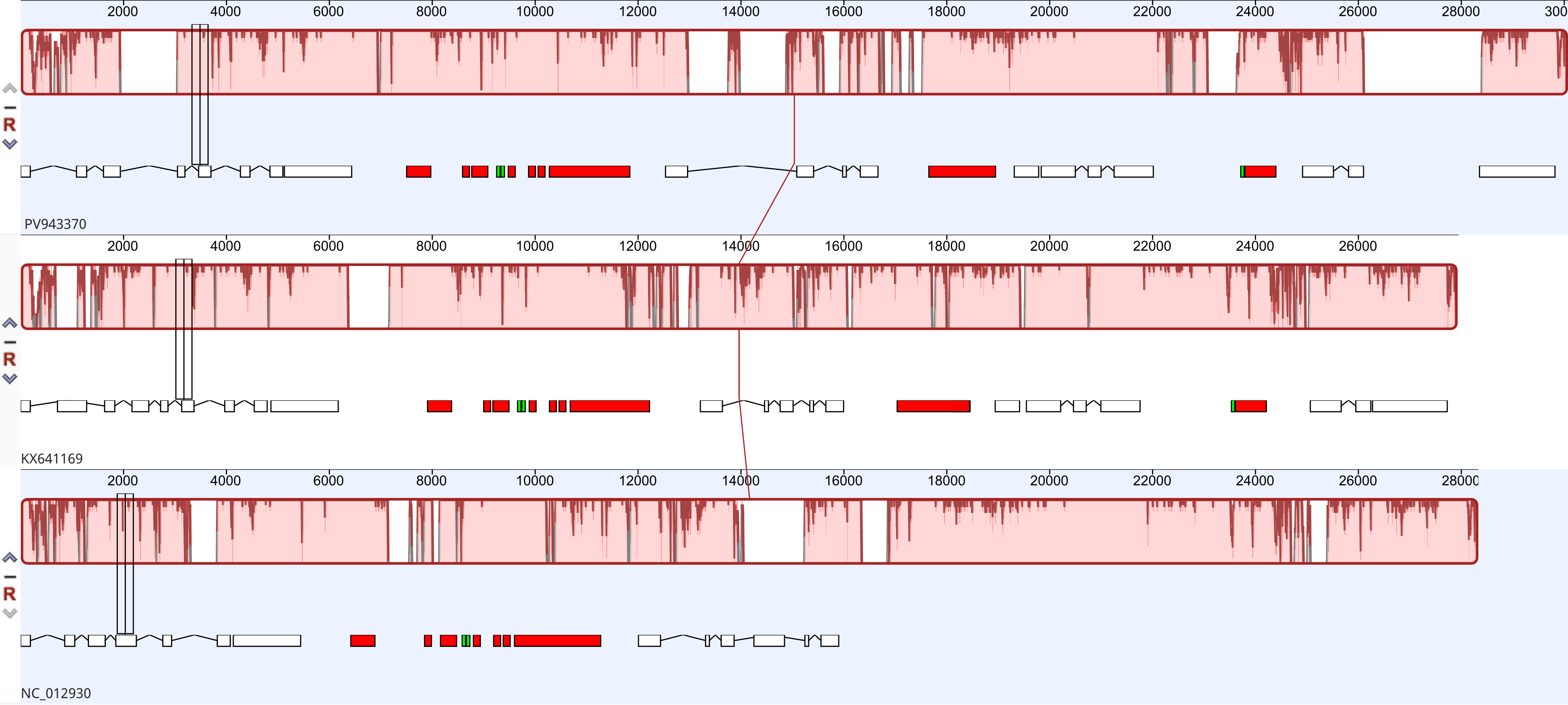


Figure 3. Mauve alignment of *Dunaliella salina* mitochondrial genomes. *Whole-mitochondrial genome alignment of* *D. salina* CS-265 (PV943370), *D. salina* GN (KX641169), and *D. salina* CCAP19/18 (NC_012930) showing conserved locally collinear blocks (LCBs) and structural rearrangements among strains.

# Discussion

The mitochondrial genome of *Dunaliella salina* CS-265 is characterized by a compact architecture and a reduced gene set, consistent with mitochondrial streamlining observed across the Chlorophyta (Del Vasto *et al.*, 2015; Smith & Keeling, 2015). The marked A+T bias (66.24%) aligns with compositional trends reported in *Chlamydomonas reinhardtii* and other green algae, where organellar genomes are similarly dominated by A+T-rich regions (Massoz *et al.*, 2014; Smith *et al.*, 2010).

The presence of intron-rich genes—particularly *cox1*, *cob*, *nad1*, and *nad5*—reflects conserved patterns of gene fragmentation commonly mediated by group I and II introns in chlorophyte mitochondria (Del Vasto *et al*., 2015; Fučíková *et al*., 2016; Magdaleno *et al*., 2017; Smith *et al*., 2010). In contrast, intronless genes such as *nad2, nad4* and *nad6* may indicate evolutionary intron loss or retention of structurally stable forms. This pattern is consistent across published *D. salina* genomes, except that the SQ strain retains a single intron in *nad4*. The absence of mitochondrial ATP synthase subunits and ribosomal proteins further supports gene reduction through endosymbiotic transfer or functional replacement by nuclear-encoded counterparts (Magdaleno *et al*., 2017; Smith *et al*., 2010).

The identification of only three tRNA genes suggests a reliance on cytosolic tRNA import or post-transcriptional editing, a strategy previously proposed for *Dunaliella* species (Magdaleno *et al.*, 2017; Smith *et al.*, 2010; Smith & Keeling, 2015). Together, these features highlight a reductive and specialized mitochondrial genome, shaped by both evolutionary pressure and metabolic constraints in hypersaline environments.

# Conclusion

The mitochondrial genome of *Dunaliella salina* CS-265 adds new insight into the diversity and structural evolution of chlorophyte mitochondria. Its compact size, high A+T content, and intron-rich genes reflect key features of adaptation and genome reduction in halotolerant green algae.

Data Availability Statement

The complete mitochondrial genome of *Dunaliella salina* Teodoresco 1905 strain CS-265 has been deposited in the NCBI GenBank database under accession number [PV943370](https://www.ncbi.nlm.nih.gov/nuccore/3024659087), associated with BioSample SAMN49745146. The raw sequencing reads are available in the Sequence Read Archive (SRA) under accession number SRR35566088, linked to BioProject PRJNA1099393 and BioSample SAMN51783953.

# Funding

The authors acknowledge the support of an Australian Government Research Training Program (RTP) Scholarship provided for doctoral research at Murdoch University.

# Disclosure statement

The authors report no conflicts of interest.

# Author Contributions

Lisa AK: Conceptualization, methodology, data curation, formal analysis, investigation, resources, software, visualization, validation, writing – original draft.
Reeve WG: Conceptualization, data curation, methodology, resources, software, supervision, validation, writing- review and editing.
Laird DW: Supervision, conceptualization, investigation, resources, writing-review and editing.
Chopra A: Supervision, methodology, resources, writing-review and editing.
Moheimani NR: Project administration, supervision, resources, writing-review and editing.

# References

Bushnell, B. (2014). BBMap/BBTools [Computer software]. DOE Joint Genome Institute, Lawrence Berkeley National Laboratory. <https://jgi.doe.gov/data-and-tools/bbtools/>

Capella-Gutiérrez, S., Silla-Martínez, J. M., & Gabaldón, T. (2009). trimAl: A tool for automated alignment trimming in large-scale phylogenetic analyses. *Bioinformatics, 25*(15), 1972–1973. <https://doi.org/10.1093/bioinformatics/btp348>

Cho, C. H., Park, S. I., Ciniglia, C., Yang, E. C., Graf, L., Bhattacharya, D., & Yoon, H. S. (2020). Potential causes and consequences of rapid mitochondrial genome evolution in thermoacidophilic *Galdieria* *(Rhodophyta)*. *BMC Evolutionary Biology*, *20*(1). <https://doi.org/10.1186/s12862-020-01677-6>

Darling, A. C. E., Mau, B., Blattner, F. R., & Perna, N. T. (2004). Mauve: Multiple alignment of conserved genomic sequence with rearrangements. *Genome Research, 14*(7), 1394–1403. <https://doi.org/10.1101/gr.2289704>

De Coster, W., D’Hert, S., Schultz, D. T., Cruts, M., & Van Broeckhoven, C. (2018). NanoPack: Visualizing and processing long-read sequencing data. *Bioinformatics*, *34*(15), 2666–2669. <https://doi.org/10.1093/bioinformatics/bty149>

Del Vasto, M., Figueroa-Martinez, F., Featherston, J., González, M. A., Reyes-Prieto, A., Durand, P. M., & Smith, D. R. (2015). Massive and widespread organelle genomic expansion in the green algal genus *Dunaliella*. *Genome Biology and Evolution*, *7*(3), 656–663. <https://doi.org/10.1093/gbe/evv027>

Félix-Castro, M. B., Arredondo-Vega, B. O., Rojas, M., & Gómez-Anduro, G. A. (2023). Synthesis of β-carotene in *Dunaliella*: From genome analysis to metabolic engineering. *Algal Research, 72.* Elsevier B.V. <https://doi.org/10.1016/j.algal.2023.103135>

Fučíková, K., Lewis, P. O., González-Halphen, D., & Lewis, L. A. (2016). Gene arrangement convergence, diverse intron content, and genetic code modifications in mitochondrial genomes of Sphaeropleales (Chlorophyta). *Genome Biology and Evolution, 8*(6), 2170–2180. <https://doi.org/10.1093/gbe/evu172>

GraphPad Software LLC. (2025). Geneious Prime (Version 2025.1.2) [Computer software]. <https://www.geneious.com>

Greiner, S., Lehwark, P., & Bock, R. (2019). OrganellarGenomeDRAW (OGDRAW) version 1.3.1: Expanded toolkit for the graphical visualization of organellar genomes. *Nucleic Acids Research*, *47*(W1), W59–W64. <https://doi.org/10.1093/nar/gkz238>

Guillard, R. R. L. (1975). Culture of phytoplankton for feeding marine invertebrates. In W. L. Smith & M. H. Chanley (Eds.), *Culture of marine invertebrate animals: Proceedings of the First Conference on Culture of Marine Invertebrate Animals, Greenport* (pp. 29–60). Springer. <https://doi.org/10.1007/978-1-4615-8714-9_3>

Gurevich, A., Saveliev, V., Vyahhi, N., & Tesler, G. (2013). QUAST: Quality assessment tool for genome assemblies. *Bioinformatics*, *29*(8), 1072–1075. <https://doi.org/10.1093/bioinformatics/btt086>

Katoh, K., & Standley, D. M. (2013). MAFFT multiple sequence alignment software version 7: Improvements in performance and usability. *Molecular Biology and Evolution, 30*(4), 772–780. <https://doi.org/10.1093/molbev/mst010>

Kalyaanamoorthy, S., Minh, B. Q., Wong, T. K. F., von Haeseler, A., & Jermiin, L. S. (2017). ModelFinder: Fast model selection for accurate phylogenetic estimates. *Nature Methods*, 14(6), 587–589.
<https://doi.org/10.1038/nmeth.4285>

Kolmogorov, M., Yuan, J., Lin, Y., & Pevzner, P. A. (2019). Assembly of long, error-prone reads using repeat graphs. *Nature Biotechnology*, *37*(5), 540–546. <https://doi.org/10.1038/s41587-019-0072-8>

Lantz, H., Dominguez Del Angel, V., Hjerde, E., Sterck, L., Capella-Gutierrez, S., Notredame, C., Vinnere Pettersson, O., Amselem, J., Bouri, L., Bocs, S., Klopp, C., Gibrat, J. F., Vlasova, A., Leskosek, B. L., Soler, L., & Binzer-Panchal, M. (2018). Ten steps to get started in genome assembly and annotation. *F1000Research, 7*, 148. <https://doi.org/10.12688/f1000research.13598.1>

Lee, R. W., Dumas, C., Lemieux, C., & Turmel, M. (1991). Cloning and characterization of the *Chlamydomonas moewusii* mitochondrial genome. *Molecular & General Genetics (MGG), 226,* 53–58. <https://doi.org/10.1007/BF00293821>

Li, H. (2018). Minimap2: pairwise alignment for nucleotide sequences. *Bioinformatics*, *34*(18), 3094–3100. <https://doi.org/10.1093/bioinformatics/bty191>

Magdaleno, D., Lopez, H., & Stephano Hornedo, J. L. (2017). The complete mitochondrial genome of the green microalga *Dunaliella* *salina* strain SQ. *Mitochondrial DNA Part B: Resources, 2*(1), 311–312. <https://doi.org/10.1080/23802359.2017.1331331>

Massoz, S., Larosa, V., Plancke, C., Lapaille, M., Bailleul, B., Pirotte, D., Radoux, M., Leprince, P., Coosemans, N., Matagne, R. F., Remacle, C., & Cardol, P. (2014). Inactivation of genes coding for mitochondrial ND7 and ND9 complex I subunits in *Chlamydomonas reinhardtii*: Impact of complex I loss on respiration and energetic metabolism. *Mitochondrion, 19*, 365–374. <https://doi.org/10.1016/j.mito.2013.11.004>

Nguyen, L.-T., Schmidt, H. A., von Haeseler, A., & Minh, B. Q. (2015). IQ-TREE: A fast and effective stochastic algorithm for estimating maximum-likelihood phylogenies. *Molecular Biology and Evolution, 32*(1), 268–274. <https://doi.org/10.1093/molbev/msu300>

Olmos, J. (2024). *Dunaliella* β-carotene productivity comparison under in vitro conditions. *Current Microbiology, 81*(5), Article 23. <https://doi.org/10.1007/s00284-024-03636-6>

Oren, A. (2014). The ecology of *Dunaliella* in high-salt environments. *Journal of Biological Research (Greece), 21*, 23. <https://doi.org/10.1186/s40709-014-0023-y>

Oxford Nanopore Technologies. (2023). *Dorado basecaller* [Computer software]. Retrieved from <https://github.com/nanoporetech/dorado>

Porebski, S., Bailey, L. G., & Baum, B. R. (1997). Modification of a CTAB DNA extraction protocol for plants containing high polysaccharide and polyphenol components. *Plant Molecular Biology Reporter, 15*(1), 8–15. <https://doi.org/10.1007/BF02772108>

Smith, D. R., & Keeling, P. J. (2015). Mitochondrial and plastid genome architecture: Reoccurring themes, but significant differences at the extremes. *Proceedings of the National Academy of Sciences of the United States of America*, *112*(33), 10177–10184. <https://doi.org/10.1073/pnas.1422049112>

Smith, D. R., Lee, R. W., Cushman, J. C., Magnuson, J. K., Tran, D., & Polle, J. E. (2010). The *Dunaliella salina* organelle genomes: Large sequences inflated with intronic and intergenic DNA. *BMC Plant Biology, 10*, 83. <https://doi.org/10.1186/1471-2229-10-83>

Smith, D. R., Hamaji, T., Olson, B. J. S. C., Durand, P. M., Ferris, P., Michod, R. E., Featherston, J., Nozaki, H., & Keeling, P. J. (2013). Organelle genome complexity scales positively with organism size in volvocine green algae. *Molecular Biology and Evolution, 30*(4), 793–797. <https://doi.org/10.1093/molbev/mst002>

The Galaxy Community. (2022). The Galaxy platform for accessible, reproducible and collaborative biomedical analyses: 2022 update. *Nucleic Acids Research*, *50*(W1), W345–W351. <https://doi.org/10.1093/nar/gkac247>

Tillich, M., Lehwark, P., Pellizzer, T., Ulbricht-Jones, E. S., Fischer, A., Bock, R., & Greiner, S. (2017). GeSeq: Versatile and accurate annotation of organelle genomes. *Nucleic Acids Research*, *45*(W1), W6–W11. <https://doi.org/10.1093/nar/gkx391>

Vahrenholz, C., Riemen, G., Pratje, E., Dujon, B., & Michaelis, G. (1993). Mitochondrial DNA of Chlamydomonas reinhardtii: the structure of the ends of the linear 15.8-kb genome suggests mechanisms for DNA replication. *Current Genetics, 24.* <https://doi.org/10.1007/BF00351798>

Wang, Y., Zhao, Y., Bollas, A., Wang, Y., & Au, K. F. (2021). Nanopore sequencing technology, bioinformatics and applications. *Nature Biotechnology, 39*(11), 1348–1365. <https://doi.org/10.1038/s41587-021-01108-x>
